# Expanding Diversity and Common Goal of Regulatory T and B Cells. II: In Allergy, Malignancy, and Transplantation

**DOI:** 10.1007/s00005-017-0471-9

**Published:** 2017-05-03

**Authors:** Grażyna Korczak-Kowalska, Anna Stelmaszczyk-Emmel, Katarzyna Bocian, Ewelina Kiernozek, Nadzieja Drela, Joanna Domagała-Kulawik

**Affiliations:** 1 Department of Immunology, Faculty of Biology, University of Warsaw, Warsaw, Poland; 2 Department Pneumonology, Medical University of Warsaw, Warsaw, Poland; 3 Department of Laboratory Diagnostics and Clinical Immunology of Developmental Age, Medical University of Warsaw, Warsaw, Poland; 4 Department of Clinical Immunology, Transplantation Institute, Medical University of Warsaw, Warsaw, Poland

**Keywords:** Regulatory T cell, Regulatory B cell, Allergy, Cancer, Transplantology, Myeloid cells

## Abstract

Regulation of immune response was found to play an important role in the course of many diseases such as autoimmune diseases, allergy, malignancy, organ transplantation. The studies on immune regulation focus on the role of regulatory cells (Tregs, Bregs, regulatory myeloid cells) in these disorders. The number and function of Tregs may serve as a marker of disease activity. As in allergy, the depletion of Tregs is observed and the results of allergen-specific immunotherapy could be measured by an increase in the population of IL-10^+^ regulatory cells. On the basis of the knowledge of anti-cancer immune response regulation, new directions in therapy of tumors are introduced. As the proportion of regulatory cells is increased in the course of neoplasm, the therapeutic action is directed at their inhibition. The depletion of Tregs may be also achieved by an anti-check-point blockade, anti-CD25 agents, and inhibition of regulatory cell recruitment to the tumor site by affecting chemokine pathways. However, the possible favorable role of Tregs in cancer development is considered and the plasticity of immune regulation should be taken into account. The new promising direction of the treatment based on regulatory cells is the prevention of transplant rejection. A different way of production and implementation of classic Tregs as well as other cell types such as double-negative cells, Bregs, CD4^+^ Tr1 cells are tested in ongoing trials. On the basis of the results of current studies, we could show in this review the significance of therapies based on regulatory cells in different disorders.

## Regulation of Immune Response in Allergy

Various abnormalities in regulatory network have long been considered as the main step in development and maintenance of allergic diseases. Regulatory T cells (Tregs) are extremely important part of this process. Among others they inhibit the production of interleukin (IL)-4, IL-5, IL-9, and IL-13 by direct suppression of Th2 cells activation, induce IgG4 instead of immunoglobulin E (IgE) production in B cell, and block the migration of effector T cells into inflamed tissue (Baecher-Allan et al. 2004; Kleer et al. 2004; Palomares et al. 2010). Frequency and functional deficiency of Tregs can be caused by any factor that disturbs immunological balance, such as environmental factors or genetic predisposition (Lambrecht and Hammad 2013). However, Tregs are not the only regulatory cells, which have a role in maintaining allergen tolerance, and if impaired lead to developing allergy. Recently, also B regulatory cells (Bregs) have been described. Like Tregs, Bregs have also a variety of mechanisms of suppression. Their most common mechanism of action is based on IL-10 production and secretion. Other regulatory function can be carried out through transforming growth factor (TGF)-β, Granzyme-B, Fas ligand production, or mediating anti-inflammatory mechanism due to their capacity of inhibitory IgG4 and sialylated IgG production (Braza et al. 2014). Certainly, they are important for the establishment of allergen tolerance (van de Veen et al. 2013).

Many authors agree that during allergy the number of Tregs is often decreased and their function is impaired (Akdis et al. 2004; Lee et al. 2007; Stelmaszczyk-Emmel et al. 2013; Xu et al. 2007). Nevertheless, the role of Tregs in the pathogenesis of pediatric allergic disorders is still unclear and the results obtained from many studies are inconsistent. A large group of authors demonstrated the reduction of the Treg population often accompanied by the impairment of their function (Lee et al. 2007; Stelmaszczyk-Emmel et al. 2013; Xu et al. 2007; Zhang et al. 2008). However, other scientists showed that the frequency of Tregs does not differ between allergic and non-allergic populations (Geraldes et al. 2010; Grindebacke et al. 2004; Ling et al. 2004). The observed discrepancies may have several reasons, including differences in clinical form of the disease, age of the patients, exposure to allergens, drugs used during the therapy (e.g., higher frequency of Tregs during steroid therapy was observed) (Karagiannidis et al. 2004; Lee et al. 2007; Shi et al. 2004). Other important factors, which influence the results include laboratory techniques used for Treg identification, the way of their characterization by different surface and intracellular markers, and the type of the collected clinical materials (peripheral blood, bronchoalveolar lavage fluid, sublingual epithelium or nasal mucosa) (Hartl et al. 2007; Nguyen et al. 2008, 2009; Radulovic et al. 2008; Scadding et al. 2010; Thunberg et al. 2010). There are still many inconsistencies in Breg phenotyping: they are characterized differently by authors, but greater agreement with regard to the direction of changes during allergy can be observed. So far, only reduction of Breg number in peripheral blood of allergic patients was observed (Kamekura et al. 2015; Kim et al. 2016; Noh et al. 2012; Stanic et al. 2015; van de Veen et al. 2013; van der Vlugt et al. 2014).

In addition to a lower number of Tregs, some authors also demonstrated the relationship between the severity of the disease and proportion of Tregs (Lee et al. 2007; Meszaros et al. 2009; Shi et al. 2004). Our studies have shown a strong correlation between relative number of Tregs and clinical manifestation of the disease. Children with characteristic symptoms of respiratory tract allergy (controlled asthma, allergic rhinitis, and allergic conjunctivitis) and additionally coexisting atopic dermatitis and/or food allergy had significantly lower percentage of Tregs in their peripheral blood than children without additional symptoms (Stelmaszczyk-Emmel et al. 2013).

Allergen-specific immunotherapy (ASIT) is nowadays the best and non-symptomatic allergy treatment. Regardless of the type of treatment (subcutaneous, sublingual, oral), ASIT influences the function of many cells: monocytes, B cell, basophils, eosinophils, mast cells, and last but not least T cells. Speaking of T cells, except reducing allergen-specific T cell proliferation, reducing tissue Th2 cytokine production, and increasing tissue Th1 cytokine release, ASIT induces functional Tregs (Akdis and Akdis 2011; Moingeon 2013; Novak et al. 2011; Ring and Gutermuth 2011). Nonetheless, the precise mechanism of ASIT is unknown and it is difficult to assess the treatment benefit in context of Tregs.

Clinical scales to assess the treatment efficiency, such as visual analog scale, medication score, and quality of life questionnaire, are widely used. However, these scales are not always objective and may not give trustworthy results. They require huge patient’s involvement and compliance, and are not easy to perform especially in children. Moreover, the results of ASIT from year to year can be affected by duration and intensity of exposure to allergens, which is never alike between allergic seasons. It would therefore be very helpful if physicians could rely on laboratory tests that would indicate whether a patient is responsive to immunotherapy or not. Nowadays, laboratory monitoring of ASIT can be performed using several test (e.g., specific IgE serum levels, specific serum IgG4 levels, allergen-induced basophils CD63 expression, allergen-specific T cells identify by MCH class II/peptide tetramers), but none of them is sufficient and each has some limitations. Some years ago, Tregs appeared to be a good marker for such assessment. In many studies, clinically successful ASIT went together with increased proportion of Tregs, increased population of IL-10^+^ cells, and/or hypomethylation of forkhead box P3 (Foxp3) (Fujimura et al. 2011; Lou et al. 2012; Scadding et al. 2010; Sørensen et al. 2013; Suárez-Fueyo et al. 2014; Swamy et al. 2012; Syed et al. 2014). However, other authors did not demonstrate any alterations in the number of Tregs after ASIT (Kim et al. 2011; Lou et al. 2012; Moed et al. 2013; Schubert et al. 2009; Stelmaszczyk-Emmel et al. 2015; Thunberg et al. 2010). And again, typical inconsistencies between different studies (mainly caused by differences in definition of Tregs and method used to identify them, different clinical conditions, etc.) make them difficult to compare. To sum up, the majority of authors agree that efficacy of ASIT does not depend on Foxp3 Tregs, but on IL-10-producing cells, and finally, only IL-10-producing cells were considered as biomarker for ASIT monitoring (Fujimura et al. 2011). Our findings are in accordance with studies in which similar numbers of Tregs before and after treatment were observed. Similarly to the variations in the onset of allergy, the differences between patients with different manifestations of allergy were observed. Small group of patients with additional clinical symptoms (atopic dermatitis, food allergy) showed significant increase of Foxp3 Tregs after ASIT, while in the entire group of patients such phenomenon was not shown (Stelmaszczyk-Emmel et al. 2015).

In short, in the view of a large number of studies Tregs and Bregs influence many immune cells, which participate in the development of allergic reactions. During ASIT, Tregs help inactivate these cells and, as a consequence, reduce severity of patients’ symptoms and improve their quality of life.

## Regulation in Malignancy and Therapeutic Options

The recognition of the mechanisms of anti-cancer immune response opened the era of successful cancer immunotherapy. The goal of this immunomodulatory treatment in malignancy is to renew own host defense mechanisms (Aerts and Hegmans 2013). The inhibition of immune regulatory processes is a very important and promising pathway in the immunomodulatory treatment of solid tumors. Here we focus on the possibility of the silencing of the function of Tregs, macrophages polarized to the regulatory population, myeloid-derived suppressor cells (MDSCs), and some suppressive cytokines: IL-17, IL-10, and TGF-β.

### Regulatory T Cells

An increased number of Tregs within peripheral blood, lymph nodes, and tumor-infiltrating lymphocytes (TIL) observed in cancer patients were found to be an important negative prognostic factor. There is a growing body of evidence that Tregs are an ideal target for therapy improving an anti-cancer response. The mechanisms contributing to an elevated number of Tregs are as follows: the migration to the tumor site of the cells de novo arising in lymph nodes and the differentiation under the influence of mediators in the tumor environment (TME) (Gallimore and Simon 2008). The main function of Tregs is to inhibit T effector cells: CD4^+^ and CD8^+^ lymphocytes, dendritic cells (DCs), and natural killer (NK) cells in the site of immune response (Chaput et al. 2007; Orentas et al. 2006; Woo et al. 2002). In contrast to solid tumors, the role of Tregs in lymphoproliferative disorders is opposite—the simplified explanation is that these cells suppress proliferating B cells (Grygorowicz et al. 2016).

The function of Tregs in malignancy is associated with overexpression of some molecules (Baecher-Allan et al. 2004; Orentas et al. 2006). These molecules are also the markers of Tregs. These are Foxp3, cytotoxic T lymphocyte antigen-4 (CTLA-4), glucocorticoid-induced TNF receptor (GITR; CD357), and lymphocyte-activation gene 3 (LAG-3). Foxp3 is known *per se* as a negative prognostic factor in solid tumors. Evaluation of immune cell infiltrates (so-called “immunoscoring”) has shown that the increased expression of Foxp3 in lymphocytes or in tumor cells and an increased Foxp3/CD8^+^ ratio are related to tumor progression (Petersen et al. 2006). On the other hand, the presence of Foxp3-positive lymphocytes in lymphoproliferative disorders is associated with a better prognosis (Tzankov et al. 2008). It was found that malignant B cells die after contact with CD4^+^/Foxp3^+^ cells.

A very strong inductor of Tregs is CTLA-4 molecule also known as a strong suppressor of the T effector cell (Teff) function (Avogadri et al. 2011). This antigen is presented on Tregs mainly as an intracellular domain. CTLA-4 is required for Treg-mediated suppression of immune response (Krummey and Ford 2014) and the inhibitory function of CTLA-4 seems to be stronger than that of Foxp3. Tregs lose their function when the expression of CTLA-4 is reduced (Krummey and Ford 2014; Walker and Sansom 2015). CTLA-4 blockade on Teff cells is capable of activating an antitumor response and has been used recently in some solid tumor therapy (Avogadri et al. 2011; Mocellin and Nitti 2013). Thus, by blocking CTLA-4 on Tregs an additional therapeutic effect of this kind of immunotherapy could be achieved. There are two domains of CTLA-4: extracellular and intracellular. The extracellular domain is required for cell function (Tai et al. 2012). CTLA-4 traffic and the expression of this molecule are modified by the tumor environment. We observed the difference in CTLA-4 cellular distribution in lung cancer: the ratio of surface to the intracellular expression of CTLA-4 was higher in TME when compared to peripheral blood (Kwiecien et al. 2017). GITR is constitutively expressed on Tregs similarly to CTLA-4 and the persistent expression of this molecule in the tumor environment was demonstrated (Avogadri et al. 2011). The agonistic anti-GITR monoclonal antibody (mAb) suppresses Tregs and is a promising direction of therapy (Nishikawa and Sakaguchi 2010).

**Fig. 1:**
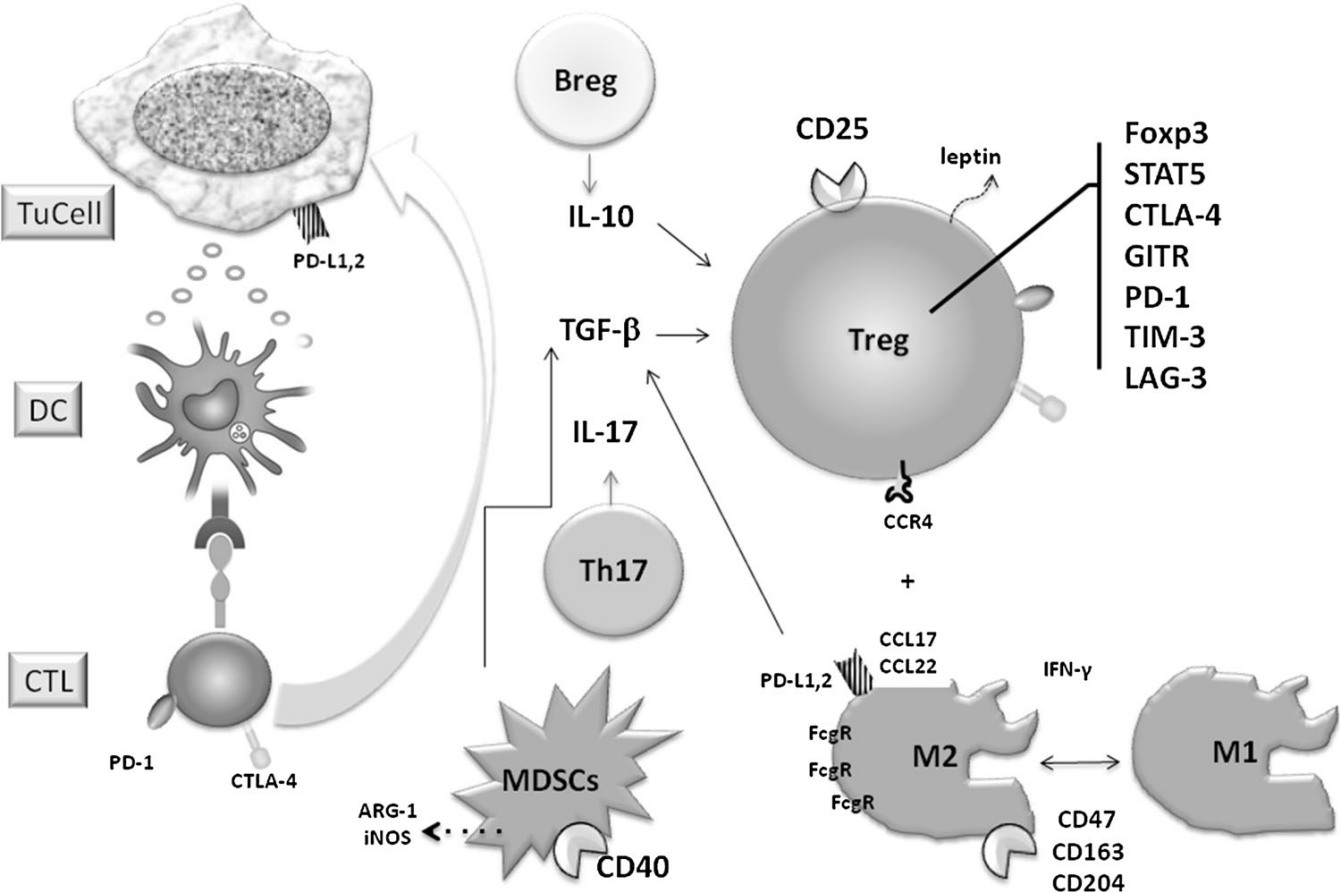
The possible targets for solid tumor immunotherapy inhibiting suppressive function of regulatory cells: Tregs, Breg, MDSCs, M2. The cytotoxic attack (on the *left*) is inhibited by cells and mediators presented on the *right*. The full explanation of reactions is presented in the text

The suppressive molecules, CTLA-4, programmed cell death protein-1 (PD-1), mucin domain containing molecule-3 (TIM-3), and the so-called “check-points,” are expressed on Teff cells and play a role of strong regulators of anti-cancer cytotoxicity. The check-point blockers anti-CTLA-4—ipilimumab and anti PD-1 nivolumab are approved in the treatment of melanoma and non-small cell lung cancer (Postow et al. 2015). PD-1 being expressed on Tregs is known to induce their suppressive and regulatory function. LAG-3 and TIM-3 play a similar role and are also the possible targets for blockade. Thus, the anti-check-point agents which are capable of restoring the anti-cancer function of cytotoxic T lymphocytes (CTLs) are simultaneously the inhibitors of Tregs (Fig. 1).

Tregs are defined by expression of CD25 (α chain IL-2 receptor), which is a possible target for Treg inhibition (Wolf et al. 2015). A classic way of CD25 blockade is to use anti-CD25 mAb. CD25 antibody—daclizumab, approved in humans in transplanthology was investigated in many cancers, but without spectacular promising results. Another method of anti-CD25 action is the use of IL-2 conjugated with diphtheria toxin (denileukin diftitox, ONTAK). The possible reason for the low efficacy of these agents is their opposite effect on Teffs and Tregs which depends on the current immune status of the tumor milieu and the stage of the maturation of targeted cells (Nishikawa and Sakaguchi 2010).

Another possible method for depleting Tregs is to modify their homing mediated by chemokines in the tumor environment. Tregs are recruited to the tumor site by the interaction of CCR4 expressed on highly immunosuppressive cells with CCL17 and CCL22 secreted by DCs and expressed by M2 macrophages (Komohara et al. 2016; Nishikawa and Sakaguchi 2010). The chemokine blocking agents causing Treg depletion in the tumor site are investigated (Kurose et al. 2015). For example, the cancers expressing NY-ESO-I testis antigen are a good model of immunogenicity and immune reaction (Nishikawa and Sakaguchi 2010). The efficacy of anti-CCR4 mAb in this model was observed (Wolf et al. 2015). It is suspected that Tregs are removed by macrophages via antibody-dependent cellular cytotoxicity (ADCC). Ipilimumab causes ADCC by mediating macrophage Fcγ receptors (FcγR) activation (Romano et al. 2015).

Some role of the pathway of arachidonic acid-cyclooxygenases (COXs)-prostaglandins in regulation of immune response in malignancy was described (Liu et al. 2015; O’Callaghan and Houston 2015). The most important is COX-2 alteration and a massive production of PGE-2 was found in the milieu of many cancers. PGE-2 is capable of inhibiting NK cells, reduce maturation of DCs, and enhance production of IL-10. The contribution of PGE-2 in the differentiation of T cells to Tregs was presented (Mougiakakos et al. 2010). Thus, the use of selective COX-2 inhibitors may have some protective anti-cancer properties and the reduction of the risk of some solid tumors was noted (Göbel et al. 2014). The targets for PGE-2 signals are four plasma membrane EP receptors: EP1-4. Each of them is connected with special cellular pathway (O’Callaghan and Houston 2015) and recently EP antagonists seem to be attractive suppressors of tumor growth in preclinical studies.

A targeted therapy using tyrosine kinase inhibitors (IKTs) proved to be very effective in patients with lung adenocarcinoma and activated epidermal growth factor receptor (EGFR) mutations. It was shown that some intracellular pathways in Tregs are also susceptible to IKTs (Wolf et al. 2015). For instance, IKTs are the inhibitors of signal transducers and activators of transcription (STAT) signaling necessary for Foxp3 expression. Other intracellular signaling pathways in Tregs are PI3 K/Akt and mTOR and may be inhibited by IKTs.

There are other studies pointing at some new anti-Treg activities that were revealed, but they need a more detailed investigation. These are the new directions:

- influencing the metabolic pathways: the suppression of Treg proliferation may be achieved by binding leptin, an adipocyte-derived cytokine (De Rosa et al. 2007),
- the evaluation of KRAS mutation which contributes to the induction of Tregs (Zdanov et al. 2016), and
- the measurement of hypoxia which promotes the suppression of immune response (Labiano et al. 2015).

Interestingly, the possible favorable role of Tregs in cancer development is considered. Persistent inflammation is a risk of malignant transformation of epithelial cells of many organs such as lung, bowel, stomach. The role of colon microbiome in chronic inflammation in relation to carcinogenesis was described in the studies on colorectal carcinoma (Feng et al. 2015). Tregs may have a protective anti-cancer role as the cells which contribute to control chronic inflammation. We previously presented the depletion of Tregs in chronic obstructive pulmonary diseases (COPD) (Domagala-Kulawik et al. 2011). COPD is a chronic inflammatory pulmonary disorder and it is recognized as a risk factor for lung cancer. The depletion of Tregs may cause the development of uncontrolled inflammation and promote malignant changes of bronchial epithelium.

It should be noted that the anti-cancer immune reaction is highly individual, dynamic, and plastic. These features are exhibited mainly by cytokines. TGF-β and interleukins, such as IL-10 and IL-6, have a well-documented inhibitory and regulatory function (Burkholder et al. 2014). However, these cytokines interact with the current direction of immune reaction and their function is strictly dependent on “of the moment” character of immune response. TGF-β is a strong suppressor, but it was demonstrated that in the initial stage of carcinogenesis TGF-β is required for the stimulation of CTLs (Quatromoni et al. 2013). The dual role of TGF-β is well visible during the differentiation of naïve Th cells to regulatory populations: the low concentration of this cytokine is capable of inducing Th17 cells, while the high concentration is capable of inducing Tregs (Romagnani 2008). The conversion of Tregs to Th-17 cells seems to be a promising way for therapeutic modification (Burkholder et al. 2014). These two populations are competitive in terms of their number, differentiation, and function. The adverse correlation between them was found in the tumor milieu; however, in our study the opposite relation was observed: the concentration of IL-17A correlated with Tregs in the lung affected by cancer (Kwiecien et al. 2017). Th17 role in malignancy remains controversial (Guery and Hugues 2015) and the high plasticity of Th17 function, dependent on IL-1β, IL-6, IL-23 concentration in the site of cancer, is known (Muranski and Restifo 2013). Recently, Punt et al. (2015) presented the impact of IL-17 and Th17 cells on the prognosis of different cancers and concluded that IL-17 was associated rather with a poor prognosis and that patients exhibiting a high IL-17 level may benefit from anti-IL-17 treatment or from the transfer of Th17 cells. Interestingly, the authors indicate the necessity to distinguish Th17 cells from other populations of IL-17-producing cells (Punt et al. 2015).

### Regulatory B Cells

Recently, a new population of cells influencing Tregs was recognized, i.e., Breg set. A deep characteristic of Bregs was presented in part I of this review. The role of Bregs in malignancy is much less defined and recognized than the role of Tregs. A very important direction and the function of Bregs confirmed in many studies is the induction of Treg generation (He et al. 2014). As the main feature of Bregs is IL-10 production, this cytokine seems to be the most important as a Treg inducer.

In the study on gastric cancer, Wang et al. (2015) found that the proportion of CD19^+^CD24^high^CD38^high^ Bregs is augmented with the capacity to produce large amounts of IL-10 and TGF-β1. These Bregs positively correlated with the levels of CD4^+^Foxp3^+^ Tregs in gastric cancer. Bregs were shown to play an immunosuppressive role in gastric cancer by inhibiting Th cytokine production, but also by converting CD4^+^CD25– effector T cells to CD4^+^Foxp3^+^ Tregs in a TGF-β1-dependent way (Wang et al. 2015). Biragyn et al. (2014) described the phenotype and function of tBregs—tumor-evoked Bregs. These tBregs are CD19^+^ B cells that express CD25^+^CD81^high^B7H-1^high^ and CD20^low^4-1BBL^low^ and a constitutively active transcription factor STAT3. Zhou et al. (2014) in the study on a large group of lung cancer patients found an elevated content of Bregs defined as CD19^+^CD24^high^CD27^+^ in the blood of the patients when compared to healthy subjects. It was enhanced by direct contact with lipopolysaccharide-stimulated cancer cells (Zhou et al. 2014). To date, there are no defined anti-Breg therapeutic options in malignancy. It seems that similarly to Tregs by targeting IL-10 and TGF-β, some important Breg markers such as CD19 and CD27 will contribute to inhibiting the cancer-promoting Breg function.

### Myeloid Cell Contribution

Regulatory lymphocytes work in a strict connection with other non-lymphoid cell populations. Tumor-associated macrophages form a large group of non-epithelial non-lymphoid cells in TEM. As well as “good” and “bad” lymphocytes among TIL, the distinction of macrophages to anti- and pro-cancer phenotype has been described. M1 type anti-cancer macrophages are defined upon the ability to produce pro-inflammatory cytokines. Thanks to the full repertoire of FcγR, macrophages reveal antigen-dependent cellular phagocytosis (ADCP) and are capable of eliminating tumor cells. The M2 macrophage differentiation is done under the influence of IL-4, IL-13, IL-10, and M-CSF (colony-stimulating factor). Among M2 products, there are also IL-10 and TGF-β (Martinez and Gordon 2014). The following term for M2 characteristics is used: “product of Th2 stimulation, pro-Th2 activation, immunoregulation.” Of M2 population, the M2b and M2c are suspected to be stronger immunoregulators. However, the analysis of macrophages by immunohistochemical staining reveals a transition of one form to another and a double expression of M1/M2 markers (Osinska et al. 2014). The correlation of M2 and Tregs is connected with a poor prognosis in malignancy. An important pathway is represented by high mobility group box 1 protein secreted by tumor cells and is able to enhance the regulatory function of myeloid cells. M2 contributes to the recruitment of Tregs by chemokine pathway CCR4/CCL17/CCL22 (Wolf et al. 2015). The other suppressive effect results from expression PD-L2, a ligand to PD-1 on macrophages. PD-1/PD-L2 ligation results in the inhibition of Teff and stimulation of Tregs. We showed the usefulness of CD163 marker for M2 cell identification (Osinska et al. 2014). CD163 is known to have complex function in inflammation with the prevalence of anti-inflammatory function and is down-regulated by many pro-inflammatory cytokines (Kowal et al. 2011). Not meaningless is the influence of hypoxia on tumor progression and macrophages belong to cell populations hypoxia-dependent (Labiano et al. 2015). Hypoxia changes metabolic pathways by the activity of hypoxia inducible factor, which mediates the suppressive cellular response (Doedens et al. 2010).

Macrophages, in contrast to lymphocytes, are much less explored as a target to immunotherapy. One of the therapeutic options targeting regulatory macrophages is the stimulation of phagocytosis after the targeted therapy. The binding of antibody to tumor cell causes the stimulation of FcγR and enhances phagocytosis. This process was confirmed in leukemia (rituximab) and in solid tumors (anti-HER-2, EGFR treatment) (Weiskopf and Weissman 2015). In addition, in patients with malignancy the FcγR polymorphism causes an increased response to antibody affected tumor cells as it was shown in leukemia, breast cancer (trastuzumab), and colon carcinoma (cetuximab) (Tamura et al. 2011; Weng and Levy 2003). CD47 is a marker on tumor cells, which protects from phagocytosis and is upregulated in many cancers. Thus, anti-CD47 antibodies are able to stimulate phagocytosis being effective after confirmation of CD47 expression on tumor cells. The clinical trials with anti-CD47 antibodies are ongoing (Kong et al. 2016). The conventional anti-cancer treatment was found to improve the effectiveness of immunotherapy. The killed cancer cells are capable of activating ADCP by releasing pro-inflammatory cytokines. A new direction is engineering antibodies to facilitate binding to the receptors, such as the newly formulated anti-CD20, anti-CD19 antibodies, IgG1 variant, cytokine-conjugated antibodies (Lazar et al. 2006). The other promising achievement is blocking of M2 receptors: CD204, folate receptor β, TIM-3, and CSF-1 (Nywening et al. 2016). Some plant-derived triterpenoids exhibit strong immunoactivatory function by anti-M2/MDSCs.

MDSCs are hematopoietic cells which originate from bone marrow in different pathological disorders, among others, and in malignancies. Different phenotypes of MDSCs were described; therefore for identifying these cells, the following markers are used: CD11β, CD14, CD15, CD33, HLA-DR (Katoh and Watanabe 2015; Komohara et al. 2016; Solito et al. 2014). The persistence of MDSC population in TEM is guaranteed by mediators secreted by cancer cells. The function of MDSCs is to inhibit T cell activation, DC differentiation, and to promote Tregs by cell-to-cell contact. The expansion of Tregs occurs thanks to the expression of CD40 on MDSCs and interaction with CD40L on Tregs in the presence of TGF-β and IL-10 (Burkholder et al. 2014). A proper T cell activation and memory-type differentiation depends on arginine, cysteine, and nitric oxide usage and MDSCs inhibit immune response by competitive use of these substrates (Srivastava et al. 2012). MDSCs produce a number of radical species and suppressive cytokines, and in this way favor angiogenesis, vasculogenesis, and metastases. The effective function of these cells is provided by epithelial–mesenchymal transition (Maenhout et al. 2014; Toh et al. 2011). The anti-MDSC strategies consist of blocking of the proliferation and differentiation and of inhibition of the migration and accumulation of these cells by different unspecific agents (Draghiciu et al. 2015; Katoh and Watanabe 2015).

### Future Directions in Immunomodulatory Treatment

Malignant disease is a complex disorder, and the immune response against neoplasm is complex. There is a growing body of evidence that only combination therapy is capable of activating the host immune system most effectively. Moreover, many results of in vivo observation indicate that one agent influences many pathways. A good example is a check-point blockade which activates T effector cells while inhibiting Tregs and regulatory macrophages (Burkholder et al. 2014). The same observation concerns the combination of conventional treatment with immunotherapy. A very important role of chemo- radiotherapy is to enhance the antigenicity of tumor cells and thus the presentation of a “new victim” to antigen-presenting cells (APC) inducing cytotoxic attack. Similarly, targeted therapy is able to change the nature of malignant cells to make them well “visible” by the immune system (Galluzzi et al. 2012; Tartour and Zitvogel 2013). The rationale for combining immunotherapy with conventional chemo- and radiotherapy and targeted therapy as well is

- to achieve the induction of immunogenic cell stress and cell death,
- to achieve the induction of antigen expression and modulation antigenic repertoire, promoting antigen cross-presentation,
- to favor releasing pro-inflammatory cytokines which recruit immune cells, support DCs migration to lymph nodes, and induce death cell receptors on tumor cells, and
- to kill MDSCs and inhibit Foxp3 expression.

Moreover, regulatory/suppressor cells form an actively multiplied population when compared to effector cells and seem to be more susceptible to chemotherapy than the less numerous CTLs. It should be noted that tumor cells also play a specific role of regulators in the tumor site. They overexpress important ligands to death receptors, such as Fas ligand and PD-L1, which induce apoptosis of Fas-positive Teffs (Hoser et al. 2004). Conventional therapy is capable of destroying these pathways.

## The Role of Cell Therapies Based on Regulatory T and B Cells in Transplantology

The innate immunity response caused by tissue injury as well as adaptive immunity response resulting from immunologic incompatibility for histocompatibility antigens between donor and recipient need to be controlled in patients undergoing cell and organ transplantation.

Although macrophages, DCs, and T and B cells may participate in the destruction of transplanted cells, they may as well develop tolerance resulting in longer graft survival. The population of regulatory cells participating in the prevention of transplant rejection and graft versus host (GvH) reaction includes CD4^+^ T cells, CD8^+^ T cells, Bregs, CD4^−^CD8^−^ T lymphocytes, NKT cells, γδ T cells, regulatory macrophages, tolerogenic DC (tolDC), MDSCs, and mesenchymal stem cells (Wood et al. 2012). Of the cells listed above, a role of the Tregs has been best understood and are most commonly used in cell therapies.

In the early stages of immune response, the strength as well as the number of recipient regulatory cells (before the transplantation or generated during the response) is not sufficient to get the large number of leukocytes capable of damaging the transplant under control. The combination of immunosuppressive agents received by the patient in order to inhibit the immune response against transplantation antigens depends on the kind of transplantation and protocols used in customized programs. The most commonly prescribed combinations of immunosuppressive drugs include calcineurin inhibitors (tacrolimus or cyclosporin A) and proliferation inhibiting factor (mycophenolate mofetil, rapamycin). Both medication types inhibit the activation of effector T cells but, they may also influence the generation and function of regulatory cells (Battaglia et al. 2005; Calvo-Turrubiartes et al. 2009; Chen et al. 2000; Coenen et al. 2006; Gao et al. 2007; Ligocki and Niederkorn 2015; Safa et al. 2015; Tebbe et al. 2016). Such phenomenon was also confirmed in our observations (Bocian et al. 2010; Korczak-Kowalska et al. 2007; Korecka-Polak et al. 2016).

The use of immunosuppressive agents contributes significantly to ensure graft survival; however, it is associated with a range of side effects (Marcen 2009; van der Net et al. 2016). The cases of long-term transplant survival in humans have been reported in spite of discontinuation of immunosuppression. This applies to patients, who had their immunosuppressive therapy discontinued for clinical reasons (chronic viral infections and tumors), patients who discontinued their immunosuppressive therapy by themselves, some patients who had liver transplanted while participating in programs for the discontinuation of immunosuppressive medicaments, and finally some patients after kidney transplantation treated according to tolerance development protocols.

The limitation of therapies based on immunosuppressive agents is required to increase the length and quality of life of the recipients of cell and organ transplants. The therapies utilizing regulatory cells seem to be promising, as they may allow to reduce the demand for immunosuppression (van der Net et al. 2016). The cell therapy includes the adoptive transfer of regulatory cells, which were prepared in vitro and in vivo generation of alloreactive regulatory cells (Kitchens and Adams 2016; Pierini et al. 2016; Scalea et al. 2016).

### Regulatory T Cells

Natural thymus-derived Treg cells (tTregs, formerly nTregs) (Abbas et al. 2013) already present in the recipient’s body during transplantation procedure are transferred into the graft, where they can inhibit the ischemia-induced injuries. Moreover, the lymphatic tissue draining the transplant contains Tregs, which inhibit the proliferation of T lymphocytes. Bregs and tolDC may promote the development of peripherally derived (formerly induced) Tregs (Abbas et al. 2013) from naive T cells (van der Net et al. 2016).

Among other Tregs, the important role in such processes is attributed to Tr1 cells, CD8^+^ Tregs, and double-negative (DN) T cells (CD4^−^CD8^−^) (Wood et al. 2012).

The suppressive activity of Tregs **(CD4** ^**+**^ **CD25** ^**+**^ **Foxp3** ^**+**^ **)** results from the influence on maturation and function of APC, synthesis of suppressive cytokines, possible induction of effector cell apoptosis, and possible disruption of metabolic pathways (Juvet et al. 2014; Safinia et al. 2015).

The greatest amount of data concerning the efficacy of therapies based on Tregs in the experimental studies was obtained using mice, rat, canine, porcine, and primate monkey models. Many data indicate the beneficial clinical role of Tregs in controlling the transplant rejection and GvH disease (GvHD) (Kitchens and Adams 2016; Trzonkowski et al. 2009; van der Net et al. 2016).

Despite the fact that different regulatory cells were used in therapeutic trials, most data concern Tregs. The most important molecules allowing for the identification of Tregs are CD4, CD25^high^, Foxp3, CD127^low^, and CD45RA.

The important stage in the development of cell therapies included the identification of the markers, which would allow the isolation of Tregs for therapeutic purposes. It has been proved that CD4^+^CD25^+^CD127^low^ cells show higher suppressive activity than CD4^+^CD25^high^ cells (Nadig et al. 2010).

There are two methods of preparation of Tregs for therapeutic purposes:

1. tTregs may be isolated from the peripheral blood, proliferated ex vivo, and administered to the patients (Dieckmann et al. 2001). tTregs are isolated from the transplant recipient, proliferated in adequate conditions, according to the valid protocols. Once CD8^+^ T cells are removed, T cells are stimulated by beads coated with anti-CD3/anti-CD28 antibodies in the presence of recombinant IL-2. The medium is enriched with rapamycin to inhibit the proliferation of cells other than Tregs. This method leads to obtaining the polyclonal Treg cells (Juvet et al. 2014; Nadig et al. 2010; Peters et al. 2008; Trzonkowski et al. 2009).
2. Alloantigen peripherally derived Tregs (pTregs) may also be prepared. Allogeneic APC cells are therefore used and the culture medium is enriched with TGF-β. pTregs were shown to display higher antigen specificity and it seems that their suppressive activity is stronger or comparable to that of tTregs; however, it requires further studies (Juvet et al. 2014; Landwehr-Kenzel et al. 2014; Sagoo et al. 2011).

Regulatory cells may also develop in situ in transplant recipients, once they received medication, which affect the development of Tregs or conversion of allospecific naive T cells to pTregs. Such therapies may also support the development of CD8^+^ Tregs and other regulatory cells (Juvet et al. 2014).

Tregs are mainly utilized in the GvHD treatment and prevention, as well as in patient, who received vascularized organ grafts (Scalea et al. 2016; van der Net et al. 2016). In the currently running (since 2014), phase I/II, multicentre clinical trials (ONE Study), Tregs, as well as Tr1, regulatory macrophages, and tolDC are administered to the kidney transplant recipients. Both the effectiveness and safety of the therapy are evaluated (Ferrer et al. 2014; Geissler 2012; Gregori et al. 2012; van der Net et al. 2016).

**Type-1 Tregs** (CD4^+^ Tr1 cells) are the second important regulatory cell population within CD4^+^ cells. Their characteristic properties include the synthesis of large amounts of IL-10 and no expression of Foxp3. The co-expression of CD49b and LAG-3 is also suggested (Gagliani et al. 2013). It has been shown that these cells participate in the development of transplantation tolerance both in animal models (tolerance for pancreatic islets transplants) and in human patients (tolerance for kidney, pancreatic islets, liver, and stem cell transplants) (Ligocki and Niederkorn 2015; Zeng et al. 2015). The clinical trial of administering Tr1 cells to the patients who underwent stem cell transplantation (phase I/II) revealed that these cells induce faster immunological restoration than Tregs, which in turn inhibited mainly GvH reaction (Gregori et al. 2012).

Among the described populations of regulatory CD8^+^ T cells **(CD8** ^**+**^ **Treg)**, there are CD8^+^CD28^−^ T cells that are worth mentioning, as they inhibit the activation of T cells by promoting the development of tolerogenic DC. These cells were found in patients who had received mAb (alemtuzumab) in induction therapy following renal transplantation. CD8^+^ Tr cells, which produce IL-10, are to a large extent functionally similar to the CD4^+^ Tr1 cells (Ligocki and Niederkorn 2015; Wood et al. 2012). The significance of CD8^+^Foxp3^+^ T cells for inhibiting immunological response after allogeneic bone marrow transplantation has also been shown (Robb et al. 2012). It has been proven that the presence of CD8^+^ Tregs may increase survival of allogeneic skin, kidney, pancreatic islets, and heart grafts (Ligocki and Niederkorn 2015). However, regulatory CD8^+^ T cells have not been used in cell therapies yet.

Peripheral **αβ-TCR** ^**+**^ **CD3** ^**+**^ **CD4** ^**−**^ **CD8** ^**−**^ **NK1.1** ^**−**^ **T cells** (DN) inhibit the response of CD4^+^, CD8^+^ cells, B cells, NK cells, and DC. Due to their antigen-specific mechanism of action, they also prevent the graft rejection and GvHD (Ligocki and Niederkorn 2015; Wood et al. 2012). Most of the data stem from the animal models and suggest the importance of apoptosis as the mechanism of killing target cells by DN cells. The suppressive function of DN cells in human is reversible and does not require the induction of apoptosis of the target cell. The role of these cells in the survival of heart, skin, and pancreatic islets grafts has already been described (Ligocki and Niederkorn 2015). As it was with CD8^+^ Tregs, there are no reports of trial therapeutic administration of these cells.

### Regulatory B Cells

The synthesis of alloantibodies and ability to stimulate CD4^+^ T cells make B cells important in the process of transplant rejection, especially in its chronic form. The response to antigen results in the development of Bregs, which limit the excessive response and may participate in acceptance of the transplant by the recipient. Many phenotypes of Bregs have been described, as well as a wide range of their mechanism of action (synthesis of suppressive cytokines, cytotoxicity, secretion of anti-inflammatory antibodies, expression of receptors inhibiting the immune response, induction of other regulatory cell populations) (Durand and Chiffoleau 2015; Kim et al. 2015).

The role of IL-10 in Breg function and the role of CD19^+^CD24^high^CD38^high^ B cells in the development of tolerance after kidney transplantation have also been noticed (Durand and Chiffoleau 2015; Newell et al. 2010; Kim et al. 2015). The regulatory populations of CD19^+^CD24^high^CD27^+^ and CD19^low^Foxp3^+^ cells also participate in the development of transplantation tolerance (Segundo et al. 2013).

The role of Breg cells in the development of tolerance has been described in various experimental models (Chesneau et al. 2013; Ding et al. 2011; Durand and Chiffoleau 2015; Yan et al. 2002), as well as in patients after cell and organ transplantations (Chesneau et al. 2013, 2014; Clatworthy et al. 2009; Durand and Chiffoleau 2015; Newell et al. 2010; Pallier et al. 2010).

Clinical trials aim the deletion of B cells population followed by promotion of the development of Breg cell population at the later stage of response (Durand and Chiffoleau 2015). It is suggested that the deletion of B cells achieved with mAbs (alemtuzumab, rituximab, basiliximab, ATG) in the presence of alloantigen promotes the development of donor-specific tolerance (Ferrer et al. 2014; Segundo et al. 2013).

Studies concerning the use of Breg cells are complicated by the large number of phenotypes and mechanisms of action of the mentioned Bregs. It is suggested that Breg cells may increase graft survival time by the induction of Tregs within the body (Durand and Chiffoleau 2015; Kim et al. 2015; Lee et al. 2014). As it is in the case of Tregs, the possibility of using medication, which would in vivo promote the development of Breg cells, is being considered, as well as in vitro generation of Breg cells followed by their administration to the patients, but the data are still scarce (Ferrer et al. 2014; Nouel et al. 2014; Scalea et al. 2016). There are no reports of clinical trials concerning the administration of in vitro prepared Breg cells.

Among the mentioned regulatory lymphocytes types, the greatest and possibly the sole role in cell therapies is attributed to Tregs. The therapies based on Tregs have not been initiated until recently and are associated with a range of uncertainties. The number of cells sufficient for effective therapy, the duration of treatment, and the number of doses still remain unknown. The unanswered questions include the following: Whether patient’s own tTregs are more efficient than alloantigen-stimulated pTregs? When should the preparation procedure begin before administration? May they be frozen and stored? Moreover, it cannot be clearly determined whether the pro-inflammatory signaling in the patient’s body would not cause the administered Tregs to convert into effector T cells. Many questions emerge concerning the safety of cell therapy, its efficacy, and finally its cost. The importance of immunologic monitoring of patients undergoing cell therapy is also being emphasized (Singer et al. 2014; van der Net et al. 2016). However, it should be presumed that due to a large demand, we will continue to observe the dynamic development of such therapies.

## Abbreviations

Breg: Regulatory B cell
CCL: Chemokine ligand
CCR: Chemokine receptor
CTL: Cytotoxic T cell
CTLA-4: Cytotoxic T lymphocyte antigen-4
DC: Dendritic cell
FasL: Fas ligand
Foxp3: Forkhead box P3
GITR: Glucocorticoid-induced TNF receptor
IL: Interleukin
LAG-3: Lymphocyte-activation gene 3
M: Macrophage
MDSC: Myeloid-derived suppressor cell
PD-1: Programmed cell death protein-1
PD-L: Programmed death-ligand
STAT: Signal transducer and activator of transcription 5
TGF-β: Transforming growth factor β
TIM-3: Mucin domain containing molecule-3
Tregs: Regulatory T cell
